# Medical Extended Reality in der digitalen Notfallmedizin: Von der Ausbildung zur klinischen Anwendung

**DOI:** 10.1007/s00063-023-01095-8

**Published:** 2023-12-12

**Authors:** Thomas C. Sauter, Gert Krummrey, Wolf E. Hautz, Tanja Birrenbach

**Affiliations:** 1 Universitätsklinik für Notfallmedizin, Inselspital Universitätsspital Bern, Freiburgstr. 16c, 3010 Bern, Schweiz; 2 Medizininformatik, Berner Fachhochschule, Biel, Schweiz

**Keywords:** Virtuelle Realität, Erweiterte Realität, Medizinische Ausbildung, REBOA – Resuscitative Endovascular Balloon Occlusion of the Aorta, Virtual reality, Augmented reality, Medical education, REBOA – Resuscitative Endovascular Balloon Occlusion of the Aorta

## Abstract

**Hintergrund:**

Die Notfallmedizin steht vor der Herausforderung, mit begrenzten Ressourcen eine optimale Versorgung zu gewährleisten. Insbesondere in seltenen, aber kritischen Situationen (High-acuity-low-occurrence[HALO]-Situationen) ist fundiertes Fachwissen essenziell. Bisherige Ausbildungsansätze sind zeitlich begrenzt und ressourcenintensiv.

**Ziel der Arbeit:**

Medical Extended Reality (MXR) bietet vielversprechende Lösungsansätze. Diese Arbeit gibt einen Einblick in die verschiedenen Bereiche von MXR und zeigt am Beispiel des HALO-MXR-Konzepts die Anwendung von MXR in der Notfallmedizin.

**Ergebnisse und Diskussion:**

Die MXR umfasst Augmented Reality (AR), Virtual Reality (VR) und Mixed Reality (MR). Die AR überlagert die reale Welt mit digitalen Informationen, verbessert die Wahrnehmung und ermöglicht interaktive Elemente. Die VR erzeugt eine künstliche 3D-Umgebung, in die der Nutzer eintaucht. Die MR kombiniert reale und virtuelle Elemente. Die MXR bietet Vorteile wie ortsunabhängiges Lernen, virtuelle Betreuung und Skalierbarkeit. Sie kann jedoch bestehende Ausbildungsformate nicht ersetzen, sondern sollte in ein Gesamtkonzept eingebettet werden.

Das HALO-MXR-Konzept am Inselspital Bern beinhaltet E‑Learning, simulationsbasiertes Training in VR und den HALO-Assist-Support durch AR. Der HALO-Assist bietet rund um die Uhr AR-Unterstützung bei HALO-Prozeduren mit Kommunikation über Audio und Video mit eingeblendeten Annotationen und Flowcharts.

**Schlussfolgerung:**

Die Integration von MXR in die Notfallmedizin verspricht eine effizientere Ressourcennutzung und erweiterte Trainingsmöglichkeiten. Das HALO-MXR-Konzept zeigt, wie MXR-simulationsbasiertes Training VR und AR effektiv kombiniert und die Anwendung von HALO-Prozeduren verbessert.

## Abstract (en)

**Background:**

Emergency medicine faces the challenge of providing optimal care with limited resources. Especially in rare but critical situations (high-acuity low occurrence [HALO] situations), sound expertise is essential. Previous training approaches are time-limited and resource-intensive.

**Aim of the work:**

Medical extended reality (MXR) offers promising solutions. This article gives insight into the different areas of MXR and shows the application of MXR in emergency medicine using the HALO-MXR concept as an example.

**Results and discussion:**

MXR encompasses augmented reality (AR), virtual reality (VR) and mixed reality (MR). AR overlays digital information on the real world, enhancing perception and enabling interactive elements. VR creates an artificial three-dimensional (3D) environment in which the user is immersed. MR combines real and virtual elements. MXR offers advantages such as location-independent learning, virtual mentoring and scalability. However, it cannot replace existing training formats, but should be embedded in an overall concept. The HALO-MXR concept at Inselspital Bern includes e‑learning, simulation-based training in VR and on-site, and HALO-Assist support through augmented reality. HALO-Assist provides around-the-clock AR support for HALO procedures via audio and video communication as well as overlayed annotations, objects and flowcharts.

**Conclusion:**

The integration of MXR into emergency medicine promises more efficient use of resources and enhanced training opportunities. The HALO-MXR concept demonstrates how MXR effectively combines simulation-based training in VR and AR assist to enhance the application of HALO procedures.

## Hintergrund

In der Notfallmedizin müssen häufig unter Zeitdruck weitreichende und komplexe Entscheidungen getroffen und Interventionen durchgeführt werden. Finanzielle Ressourcenknappheit und Fachkräftemangel zusammen mit steigenden Patientenzahlen stellen die Notfallmedizin vor die große Herausforderung, mit begrenzten Ressourcen eine optimale Notfallversorgung auf hohem Qualitätsniveau zu gewährleisten. Das Fehlen einer Facharztausbildung für Notfallmedizin in Deutschland, Österreich und in der Schweiz (DACH-Region) führt hier zu einer Lücke in der Expertise, die in einem Bereich mit komplexen Anforderungen ein großes Problem darstellt. In vielen Fällen ist die Ausbildung von Ärzten, die sich für Notfallmedizin interessieren, noch mehr oder weniger eine Frage von „learning by doing“ oder basiert auf dem Dogma „see one, do one, teach one“ erschwert durch Schichtdienste und nicht planbare Arbeitsbelastung. Entsprechend zeigen Bedarfsanalysen den Wunsch nach interaktiven, zeitunabhängigen Lernformen, die moderne Formen des Wissenstransfers integrieren [12, 13].

## High-acuity-low-occurrence-Situationen

Neben zahlreichen Routineaufgaben durchbrechen in unregelmäßigen Abständen sog. High-acuity-low-occurrence(HALO)-Situationen den Alltag in der klinischen und präklinischen Notfallmedizin. Diese Situationen zeichnen sich dadurch aus, dass sie zwar selten auftreten, aber gleichzeitig kritische Maßnahmen in kurzer Zeit sicher durchgeführt werden müssen. Welche Prozeduren und Situationen in die Gruppe der HALO-Ereignisse fallen, variiert je nach Setting, d. h. von Klinik zu Klinik und von Rettungsdienst zu Rettungsdienst, aber auch je nach Ausbildungsstand. Während in kleineren Häusern und für unerfahrene Notfallmediziner:innen bereits das Legen einer Thoraxdrainage als HALO bezeichnet werden kann, sind in größeren Zentren Prozeduren wie das Legen eines temporären Schrittmachers, das Legen einer „resuscitative endovascular balloon occlusion of the aorta“ (REBOA) oder chirurgische Atemwegssicherungen entsprechend selten, hoch akut und können Unsicherheiten, Probleme und Stress auslösen.

Bei lange zurückliegendem Training werden HALO-Prozeduren unter Unsicherheit durchgeführt

Notaufnahmen und Rettungsdienste begegnen diesen HALO-Situationen bisher mit verschiedenen Trainingskonzepten, die in der Regel regelmäßige Simulationstrainings, Standard Operating Procedures und Ähnliches umfassen. Wo möglich und sinnvoll werden vereinzelt auch komplexere Trainings z. B. an Körperspendern oder Tiermodellen durchgeführt. Allerdings liegen solche Trainings in einer konkreten HALO-Situation oft schon Wochen oder Monate zurück. In der Konsequenz werden HALO-Prozeduren entweder gar nicht, nicht zeitgerecht oder unter Unsicherheit durchgeführt, was potenziell erhebliche Auswirkungen auf die Gesundheit der Patient:innen sowie das Wohlbefinden der Mitarbeiter:innen hat. Je nachdem welche Maßnahmen im lokalen Kontext als HALO-Prozeduren angesehen werden, reichen die Folgen für die Patient:innen von vermeidbaren Beeinträchtigungen (z. B. durch eine verspätet eingelegte Thoraxdrainage) bis hin zum Tod (z. B. durch eine nicht eingelegte REBOA).

Große Zentren lösen die Problematik von HALO-Situationen mit entsprechenden Bereitschaftsdiensten

Große Zentren lösen die Problematik von HALO-Situationen national und international durch die Vorhaltung entsprechender Bereitschaftsdienste. In der Fläche wird diese Möglichkeit aus nachvollziehbaren wirtschaftlichen Gründen in der Regel nicht genutzt und auch große Zentren können nicht rund um die Uhr Kompetenz in allen HALO-Verfahren sicherstellen.

Um diesen und ähnlichen Problemen zu begegnen, sind in den letzten Jahren neue technische Möglichkeiten entstanden, die sowohl die simulationsbasierte Ausbildung als auch die klinische Arbeit mit innovativen Techniken unterstützen können. Die Erweiterung der Realität, auch Medical Extended Reality (MXR) genannt, mit technischen Hilfsmitteln hat das Potenzial, die simulationsbasierte Ausbildung zu verändern, aber auch im klinischen Alltag unterstützend eingesetzt zu werden.

## Medical Extended Reality

Unter MXR versteht man allgemein die Erweiterung der Grenzen der Realität der medizinischen Praxis und Ausbildung mit technischen Hilfsmitteln durch die Schaffung immersiver und interaktiver Erfahrungen. Die MXR umfasst die Augmented Reality (AR), Virtual Reality (VR) und Mixed Reality (MR).

### Augmented Reality

Die AR überlagert die reale Welt mit digitalen Informationen und verbessert die Wahrnehmung der Umgebung durch Hinzufügen kontextbezogener und interaktiver digitaler Elemente, mit denen auch interagiert werden kann. Dabei bleibt die Verbindung mit der realen Welt erhalten. Die AR kann z. B. für die Einblendung von Vitalzeichen, Flussdiagrammen oder für Ausbildung und Training eingesetzt werden [11].

### Virtual Reality (VR)

Die VR ist eine Technologie, bei der der Benutzer mithilfe eines am Kopf getragenen Geräts (VR-Headset) in eine künstliche 3D-Umgebung eintaucht. Grundlegend für das Lernen mit VR ist die Immersion, das Eintauchen in die virtuelle Umgebung und damit das Gefühl der Präsens in der virtuellen Welt [7]. Die virtuelle Umgebung bietet Interaktionsmöglichkeiten, wie in den Händen gehaltene Controller oder zunehmend direkte Interaktion mit z. B. Hand-Tracking, die es den Nutzern ermöglicht, mit ihren eigenen Handbewegungen in die virtuelle Welt einzutauchen.

Nutzer können in Zukunft mit ihren eigenen Handbewegungen in die virtuelle Welt eintauchen

Die VR-Simulationen haben sich als nützliches und wirksames Instrument vor allem für das Training chirurgischer und technischer Fähigkeiten erwiesen [10, 14]. Sie kann jedoch auch für das Training nichttechnischer Fertigkeiten genutzt werden [3]. Die Vorteile von VR für das Training von Notfallsituationen liegen vor allem in der Skalierbarkeit, die insbesondere für die beliebige Wiederholung von risikoreichen oder ressourcenintensiven Trainingsthemen wichtig ist. Im Idealfall ist ein autonomes Training möglich. Hierbei können die Auszubildenden selbständig und zeit- und ortsunabhängige lernen. Diese Vorteile waren insbesondere auch während der Kontaktrestriktionen in der Coronapandemie sinnvoll und nützlich [2].

### Mixed Reality

Die MR verschmilzt virtuelle und reale Elemente und ermöglicht es, reale Objekte in virtuelle Umgebungen einzubinden und so virtuelle und reale Welt verschmelzen zu lassen und Nachteile, wie die eingeschränkte Haptik der VR, zu überwinden. Aktuell wird diese Technik z. B. in der Ausbildung der kardiopulmonalen Reanimation verwendet, bei der ein wesentlicher Teil des Lernziels im haptischen Erleben und der korrekten mechanischen Durchführen der Herzdruckmassage liegt und verschiedene Formen der MXR eingesetzt werden können [5, 9].

Vor dem Hintergrund der zuvor beschriebenen Herausforderungen in der Notfallmedizin kann MXR eine vielversprechende Lösung sein, um zur Bewältigung der mannigfaltigen Herausforderungen beizutragen. Zu den potenziellen Vorteilen zählen

- die Flexibilität des ortsunabhängigen Lernens,
- ein virtuelles Tutorsystem mit der Möglichkeit des Zugriffs auf Fachwissen von nahezu jedem geografischen Standort aus und
- die Skalierbarkeit von MXR-Anwendungen, die die Reichweite der Ausbildung erhöht und eine effizientere Nutzung der verfügbaren Ressourcen verspricht.

Für eine vertiefte Auseinandersetzung mit den Möglichkeiten der Nutzung von MXR in der Notfallmedizin ist der Übersichtsartikel von Wu et al. zu empfehlen, der die Anwendungsmöglichkeiten der MXR in der Notfallmedizin zusammenfasst [15]. Tang et al. zeigen in ihrem systematischen Review den aktuellen Einsatz von MXR allgemein in der Ausbildung, aber auch in der klinischen Anwendung [14].

Allen Anwendungen von MXR ist gemeinsam, dass sie kein Wundermittel sein können, um bestehende Ausbildungsformate zu ersetzen, sondern dass sie in ein Gesamtkonzept für Ausbildung und Anwendung eingebettet sein sollten. Zur besseren Illustration der vielfältigen Möglichkeiten aber auch zum Aufzeigen der Herausforderungen wird hier ein Beispiel für ein MXR-unterstütztes Curriculum vorgestellt: das HALO-MXR-Konzept.

## HALO-Assist-Konzept zur sinnvollen Anwendung von MXR

An der Universitätsklinik für Notfallmedizin (UKN) des Inselspitals Bern, dem größten Traumazentrum der Schweiz und einem der größten im DACH-Raum, wird seit dem Jahr 2020 das REBOA-Verfahren im klinischen Regelbetrieb bei lebensbedrohlichen Blutungen eingesetzt [8]. Pro Jahr werden zwischen 10 und 15 REBOA-Katheter bei polytraumatisierten Patient:innen eingebracht: Es handelt sich bei der REBOA also um eine HALO-Prozedur. Da die Indikation für REBOA selten und das Verfahren anspruchsvoll ist und die Einlage nur in äußerster Notfallsituation durchgeführt wird, gibt es keinen Raum für Fehler. Auch die bisher einzige randomisierte kontrollierte Studie zu REBOA (UK-REBOA Trail) betont die Risiken der Einlage und die Wichtigkeit der Ausbildung und Erfahrung mit der Nutzung des Tools [6]. Trotzdem ist es bis jetzt unklar, wie die Ausbildung für diese Prozedur optimal aussehen sollte [4], weshalb die Autoren ein innovatives MXR-unterstütztes Konzept vorschlagen.

### Grundlage und Rahmenbedingungen

**Figure 1:**
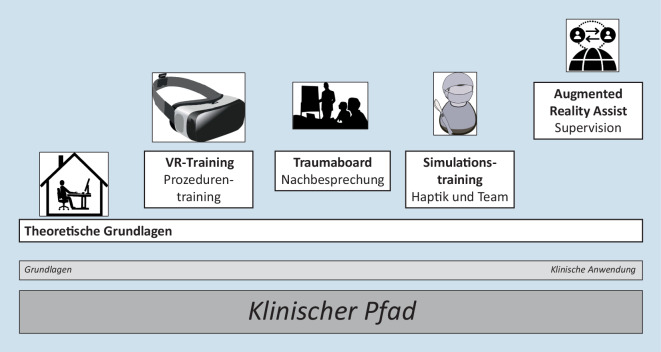

**Figure 2:**
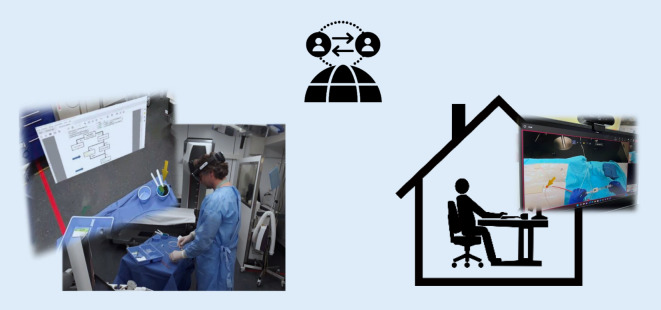

Das gesamte Konzept ist in Abb. 1 zusammengefasst und ein beispielhafter Aufbau des Augmented-Reality-HALO-Assist-Systems mit Provider im Schockraum und externem Support ist in Abb. 2 dargestellt. Eine wichtige Grundlage der Entwicklung ist die Erstellung eines klinischen Pfads unter Einbezug aller beteiligten Spezialisten, der nicht nur die Indikationen für die REBOA-Einlage, sondern auch das weitere Vorgehen nach der Einlage umfasst. Diese Theorie über die Indikation, das lokale Vorgehen und ein theoretisches schrittweises Vorgehen bei der REBOA-Implantation mit Videos wird als E‑Learning zur Verfügung gestellt. Im Rahmen der Qualitätssicherung werden zudem alle REBOA-Patient:innen und solche, die sich potenziell für dieses Verfahren qualifiziert hätten, in einem monatlich stattfindenden interdisziplinären Traumaboard besprochen.

### Simulationsbasierte Ausbildung

Ein weiterer Baustein ist der simulationsbasierte Ausbildungsteil bestehend aus VR-Training und vor Ort-Training.

Die Autoren bieten zeitunabhängig ein REBOA Training in VR an, das von ihnen speziell für das Selbststudium zusammen mit einer VR-Firma entwickelt wurde. Lernziel des VR-Trainings ist der prozedurale Ablauf, d. h. das Erlernen des schrittweisen Durchführens des Eingriffs. Die Entwicklung und Evaluation der „usability“ ist separat detailliert beschrieben [1]. Mit dem VR-Training ist ein angeleiteter Modus möglich, bei dem der Eingriff Schritt für Schritt vom virtuellen Tutor instruiert wird. Ebenfalls ist das Training durch beliebig viele Personen durchführbar und z. B. eine Instruktion durch einen erfahrenen REBOA-Provider aus den USA möglich. Das VR-Training ist zeitunabhängig verfügbar und kann ohne Trainer selbstständig durchgeführt und für Wiederholungen und Auffrischungen genutzt werden.

Die Anwendung von HALO-Assist mit Augmented-Reality-Brille wird in Simulationsübungen trainiert

Zusätzlich umfasst das Training eine vierteljährlich angebotene klassische Simulation mit haptischer Simulationspuppe zum Erfahren der echten Materialen und Erleben der Haptik. Wichtig ist hier, dass die Anwendung des im nachfolgenden beschriebenen HALO-Assist mit Augmented-Reality-Brille bereits im Rahmen von Simulationsübungen trainiert wird. Dieses Training ist nicht nur für den HALO-Assist-Provider am Patienten zum Erlernen des Umgangs mit der AR-Brille wichtig, sondern auch für den HALO-Assist-Support zu Hause, da die technischen Fallstricke, aber auch Eigenheiten der Kommunikation, wie z. B. die Gefahr der Ablenkung über die AR-Brille etc., diskutiert und trainiert werden müssen.

### HALO-Assist in Augmented Reality

Aufbauend auf das E‑Learning und die simulationsbasierte Ausbildung inklusive VR-Training wurde ein „HALO-Assist-Support“ genannter Hintergrunddienst eingerichtet, der rund um die Uhr hinzugezogen werden kann und durch Augmented Reality in der Indikationsstellung, Durchführung und Nachsorge der HALO-Prozeduren unterstützt. Aktuell ist der HALO-Assist für die Einlage einer REBOA am Inselspital seit Januar 2023 im Regelbetrieb.

Die AR-Brille stellt beim Start eine Verbindung zum HALO-Assist-Support her

**Figure 3:**
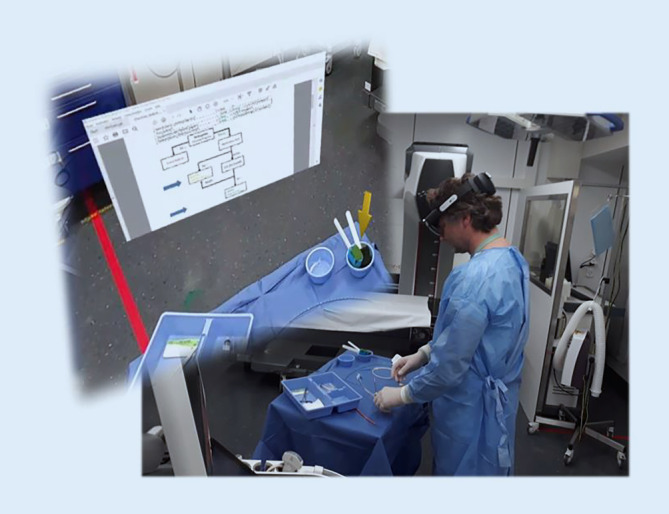

Der HALO-Assist besteht beim Provider im Schockraum aus einer HoloLens 2 (Microsoft, Redmond, WA, USA; Abb. 3) sowie einem Laptop beim assistierenden HALO-Assist-Support. Der HALO-Assist-Support wird bei Anmeldung eines hämodynamisch instabilen Traumapatienten oder einer schweren postpartalen Blutung alarmiert. Im Traumateam wird vor Eintreffen der Patient:in eine Person als HALO-Assist-Provider vor Ort bestimmt und mit der AR-Brille ausgestattet. Die AR-Brille ist ausschließlich auf mobiles Internet über ein Mobilfunknetz angewiesen und kann sowohl im Schockraum als auch präklinisch eingesetzt werden.

**Figure 4:**
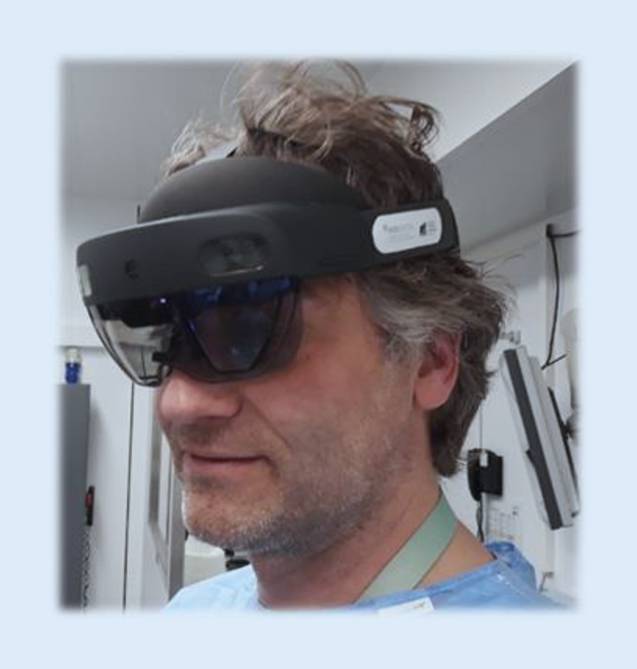

Die AR-Brille ist so konfiguriert, dass sie beim Start eine Verbindung zum HALO-Assist-Support herstellt. Dabei kommt eine Audio- und Videoverbindung analog einer normalen Videokonferenz am Computer zustande. Der HALO-Assist-Support kann sehen, was der HALO-Assist-Provider sieht und – auch in sehr lauter hektischer Umgebung – mit ihr/ihm sprechen. Daneben hat der HALO-Assist-Support die Möglichkeit, dem Provider in seinem Blickfeld eine stationäre Annotation, z. B. einen Pfeil oder eine sonstige Markierung, einzublenden oder ein Flussdiagramm anzuzeigen (Abb. 4). Der Provider selbst kann Objekte frei im Raum positionieren. Dazu erkennt die AR-Brille die Gesten des Providers. Die Bedienung funktioniert auch mit sterilen Handschuhen oder unter schwierigen Beleuchtungsbedingungen.

Die Autoren haben den Einsatz des HALO-Assists inkl. einer technischen Einführung in ihr Simulationstrainings integriert, um die Hemmschwelle zum Einsatz der Technologie zu senken und Vertrauen und Akzeptanz in die Idee des HALO-Assist und der Augmented-Reality-Technologie zu schaffen. Darüber hinaus zeichnen sie alle HALO-Prozeduren auf, um Training, Qualitätssicherung und Implementationsforschung zu ermöglichen.

Um den HALO-Assist in seiner Funktion zu unterstützen und die Dokumentation der HALO-Prozeduren zu vereinheitlichen, haben die Autoren eine „REBOA-Checkliste“ für den HALO-Assist-Support entwickelt, in der die Details der Prozedur dokumentiert werden. Insbesondere Parameter, wie Zeitpunkt des Aortenverschlusses oder Ballonvolumen, die für den weiteren Verlauf entscheidend sind, werden so zuverlässig dokumentiert.

## Ausblick

**Figure 5:**
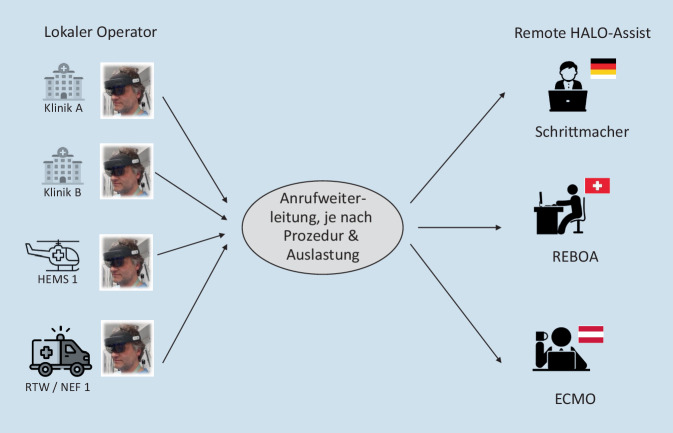

Wie bei allen MXR-Technologien kann auch beim HALO-Assist und dem VR-Training der Vorteil der Skalierbarkeit nur bei ausreichender Verbreitung voll zum Tragen kommen. Der Einsatz dieses MXR-Konzepts ist keineswegs auf eine Klinik oder die Indikation REBOA beschränkt. Vielmehr können sowohl die Anzahl der beteiligten Kliniken und Kompetenzzentren als auch die HALO-Indikationen erweitert und auf den präklinischen Bereich ausgedehnt werden. So wäre es denkbar, verfahrensspezifische Zentren an verschiedenen Standorten zu definieren (Abb. 5).

## Fazit für die Praxis

- Vor dem Hintergrund der dargestellten Herausforderungen in der Notfallmedizin, insbesondere bei der Bewältigung von High-acuity-low-occurrence(HALO)-Situationen, eröffnet die Integration von Medical Extended Reality (MXR) vielversprechende Möglichkeiten zur Bewältigung der vielfältigen Anforderungen.
- Die Flexibilität des ortsunabhängigen Lernens, die Möglichkeit der virtuellen Unterstützung von nahezu jedem geografischen Standort aus sowie die Skalierbarkeit von MXR-Anwendungen versprechen eine effizientere Nutzung der vorhandenen Ressourcen und eine Erweiterung der Reichweite der Ausbildung.
- Die vorgestellte Lösung HALO-Assist zeigt, wie MXR in der Praxis für die Lehre und klinische Anwendung umgesetzt werden kann. Durch die Kombination von E‑Learning, simulationsbasiertem Training mit Virtual Reality (VR) und dem Einsatz von Augmented Reality (AR) zur Unterstützung im klinischen Alltag wird ein ganzheitlicher Ansatz zur Verbesserung des Trainings und der Anwendung von HALO-Prozeduren verfolgt.
